# Rare metastasis of renal cell carcinoma to the breast: a case report

**DOI:** 10.1093/omcr/omaf029

**Published:** 2025-05-28

**Authors:** Aman Saswat Sahoo, Bhuvi Singh, Monther Salman, Lilia Ragad, Rasheed Elayyan

**Affiliations:** University of Central Lancashire, School of Medicine and Dentistry; University of Central Lancashire, School of Medicine and Dentistry; University of Central Lancashire, School of Medicine and Dentistry; King’s College Hospital, Breast Surgery Department; King’s College Hospital, Breast Surgery Department

**Keywords:** breast cancer, renal cell carcinoma, breast metastasis, nephrological malignancy, secondary breast neoplasm

## Abstract

Renal cell carcinoma is an aggressive urological malignancy, accounting for 2%–3% of adult cancers and over 90% of renal cancers. While renal cell carcinoma commonly metastasizes to organs such as the lungs, bones, and liver, breast metastasis is rare, comprising only 0.5% to 2% of all breast neoplasms. This case report describes a 54-year-old female with a history of RCC who developed a metastatic lesion in her breast six years after radical nephrectomy. The lesion, initially detected via routine imaging, was confirmed as renal cell carcinoma through histopathology and immunohistochemistry. A wide local excision was performed, avoiding unnecessary axillary surgery. This case highlights the need for meticulous diagnostic evaluation to distinguish between primary breast carcinoma and metastatic disease, particularly in patients with a history of renal cell carcinoma. The rarity of renal cell carcinoma metastasis to the breast calls for further research to guide appropriate management.

## Introduction

Renal cell carcinoma (RCC) is a prevalent and aggressive urological malignancy that accounts for approximately 2%–3% of adult neoplasms and more than 90% of all renal cancers [1]. RCC usually metastasizes to the lungs (70%), bones (42%), lymph nodes (55%), liver (41%), and brain (11%). Metastasis to the breast, however, is a rare phenomenon and accounts for 0.5% to 2% of all breast neoplasms [2].

Breast metastases usually originate from melanoma, lymphoma, or leukaemia making metastasis from RCC an exceptionally rare occurrence [3]. The presence of RCC in the breast indicates widespread dissemination of malignancy. The prognosis of metastatic RCC is generally poor compared to that of localised disease [4, 5]. Due to its rarity the metastasis of RCC to the breast can be overlooked for a primary breast carcinoma, hence necessitating meticulous diagnosis which will avoid extreme surgical interventions such as mastectomy.

We present herein a rare case of a 54-year-old female who presented to us with an RCC metastasis to her breast 6 years after her initial diagnosis.

## Case presentation

A 54-year-old female had a history of RCC treated Initially by radical nephrectomy and no chemotherapy.

The patient has had regular computer tomography (CT) imaging since her surgery. 3 years ago, a routine CT identified a 4 mm lesion in the lower outer quadrant of the right breast.

**Figure 1:**
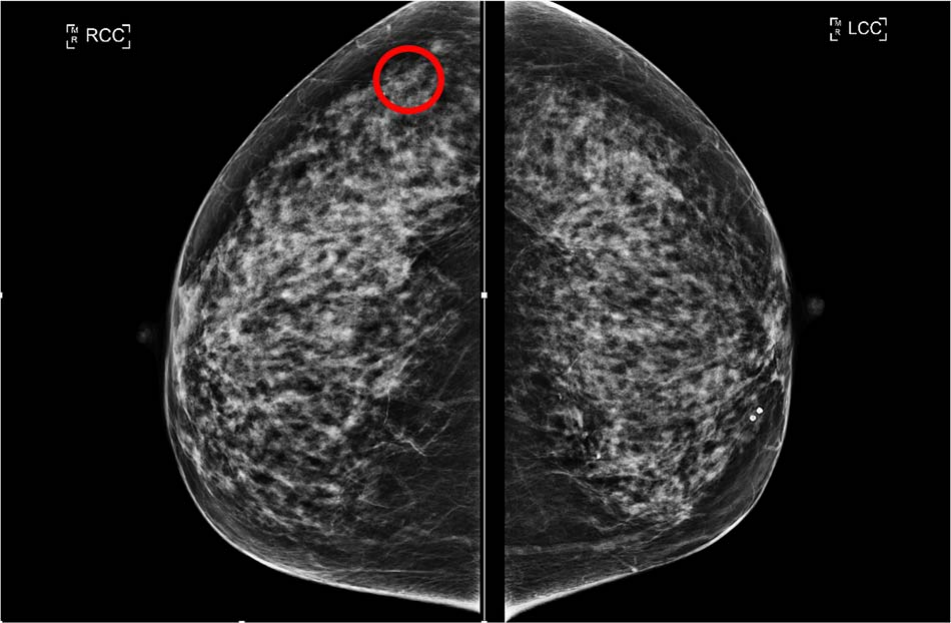
RCC (lesion circled) and LCC mammogram of the lesion.

**Figure 2:**
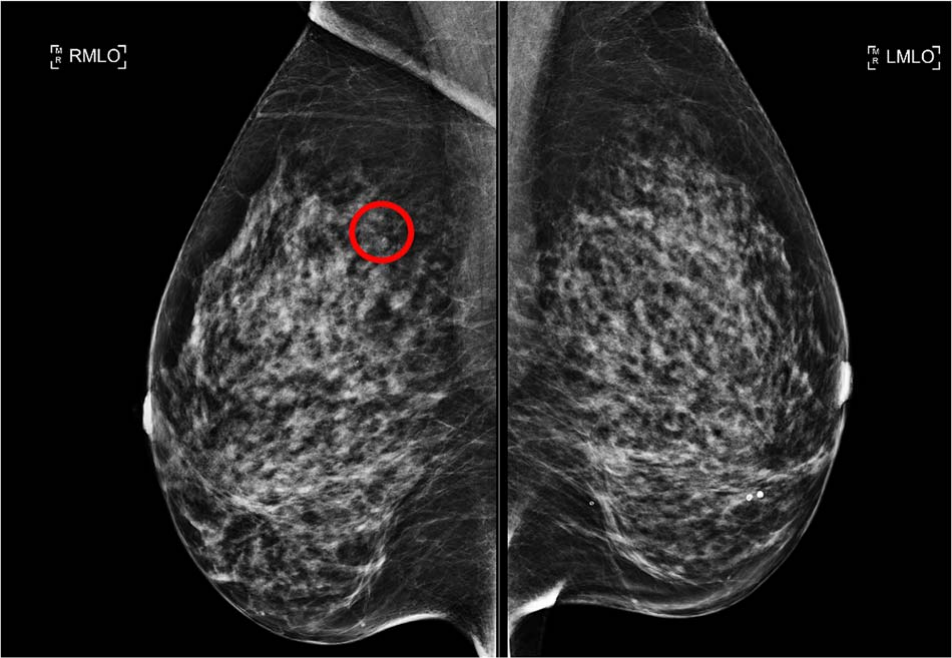
RMLO (lesion circled) and LMLO mammogram of the lesion.

Following this CT scan, a triple assessment has been performed. A clinical examination revealed no palpable abnormalities in both breasts. However, mammography highlighted a 6 mm circumscribed nodule in the posterolateral aspect of the right breast, which was not present in prior imaging 4 years ago (Figs 1 and 2).

**Figure 3:**
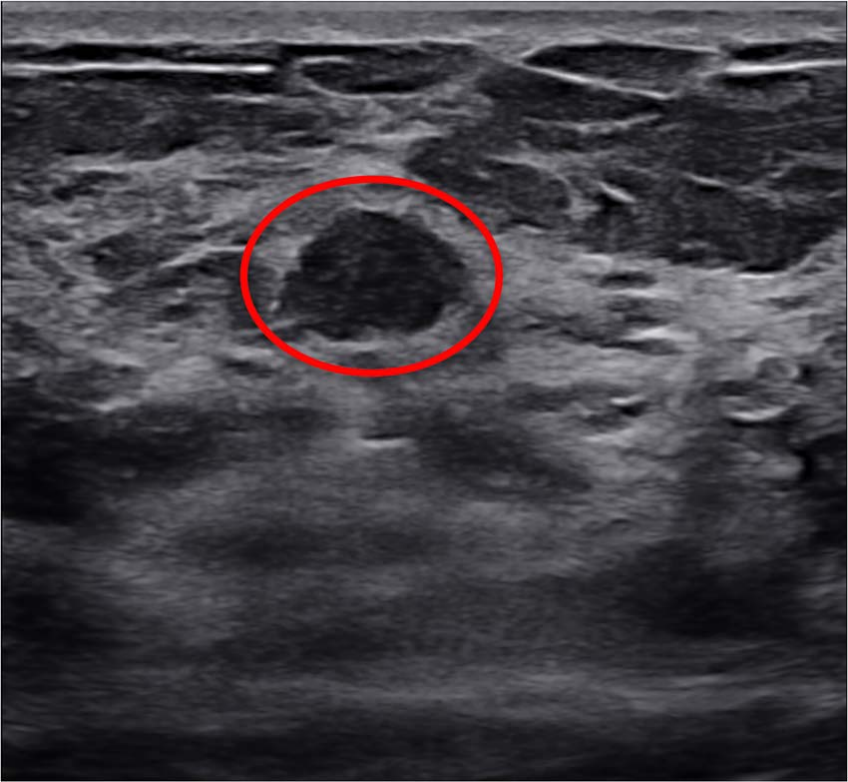
Ultrasound scan of the lesion (circled).

Ultrasound evaluation correlated these findings with a 5 mm benign-appearing nodule (Fig. 3).

Core Biopsy was performed, and histopathological evaluation showed an inflammatory lesion, characterised by cells with clear cytoplasm and macrophages. Keeping in mind the initial known diagnosis; immunohistochemical staining provided crucial diagnostic clarity, with positive results for PAX8, CD10, and MNF116 markers, confirming the presence of metastatic RCC.

The patient was informed of the diagnosis of metastatic RCC to the right breast. Further assessment of distant sites and the potential for systemic therapy were discussed at this stage; however, it was later decided that no systemic interventions would be pursued. While the lesion was considered minor in terms of surgical intervention, its identification was crucial as an indicator of metastatic disease.

**Figure 4:**
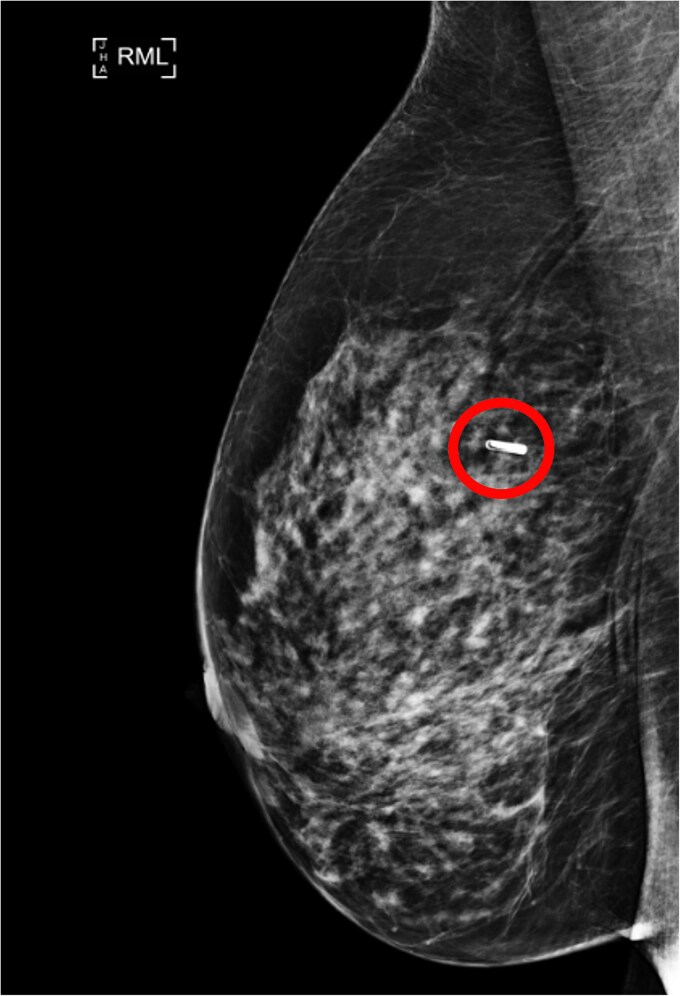
RMLO view of the localiser placement (circled).

**Figure 5:**
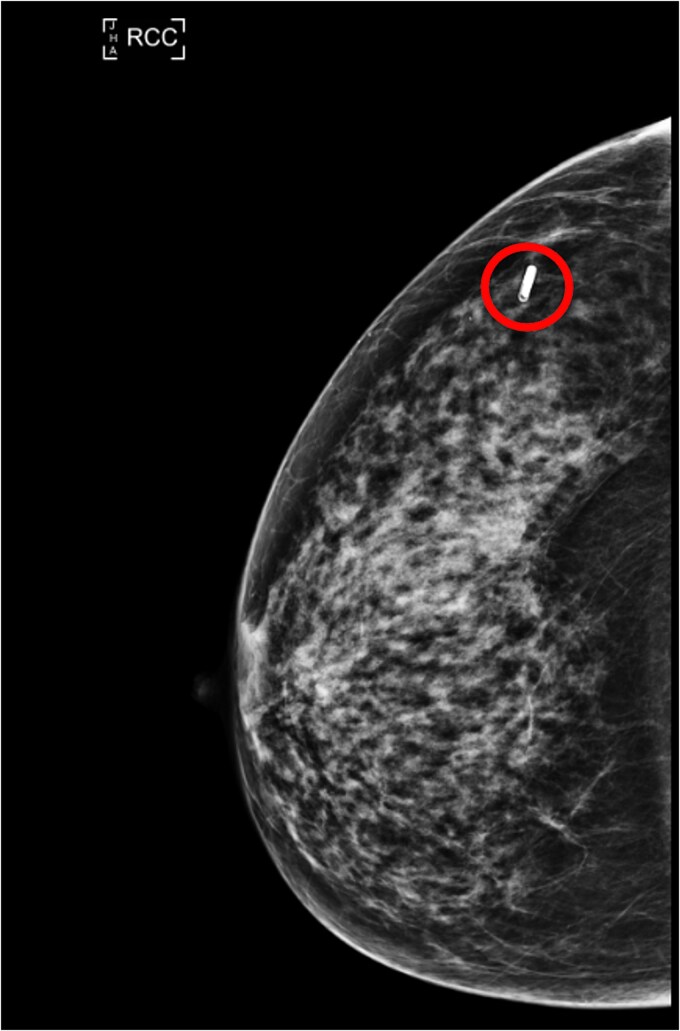
RCC view of the localiser (circled).

A wide local excision was performed after tagging the lesion with a radio frequency identification tag located 3 mm inferior to the original site; no axillary surgery was performed (Figs 4 and 5).

Post-operative Histopathological evaluation revealed a 5 mM well-circumscribed metastatic renal cell carcinoma. No vascular invasion, ductal carcinoma in situ, or lobular carcinoma in situ was identified.

## Discussion

RCC is prominent in its ability to spread hematogenously, yet metastasis to the breast is extremely uncommon [3].

The route for metastasis usually includes the migration of tumour cells from the kidneys through the inferior vena cava to the right ventricle of the heart. From here they enter the pulmonary circulation, and eventually to the breast [6].

Breast metastasis from RCC is exceedingly rare, with fewer than 60 cases documented in the literature. Although the risk of RCC recurrence is highest within the first two years following treatment, metastases to uncommon sites, such as the breast, have been reported even a decade after the initial diagnosis and surgical intervention [7]. Similarly, in our case, the metastasis was identified 8 years after nephrectomy.

Metastatic breast cancer typically presents as a rapidly growing painless firm palpable breast mass. They usually have well-defined or close to well-defined margins and are rounded in shape. Multiple tumours in one breast or bilateral tumours are rarely seen [8].

It is important to differentiate primary disease from secondary metastasis due to overlap in clinical and radiological features. A history of malignancy or co-existing tumour should raise suspicions of a secondary tumour. Differentiating between the two will guide the treatment strategy for individual patients. A triple assessment must be carried out for accurate diagnosis [9].

There are no specific guidelines for the management of RCC metastasis of the breast. Surgical excision is often used when the metastasis is isolated, as is the case with most breast metastasis. Regardless of the primary source, metastasis to the breast is associated with poor outcomes with a mean survival of 10.9 months [10].

The limited information available in the literature regarding optimal treatment strategies and patient outcomes for RCC metastasis to the breast highlights the need for further studies to better understand this condition.
